# Persistent BCR::ABL1 p190 Minimal Residual Disease and Declining Donor Chimerism Following Haploidentical Bone Marrow Transplant in Pediatric Acute Myeloid Leukemia With Dual High-Risk Fusions

**DOI:** 10.7759/cureus.96665

**Published:** 2025-11-12

**Authors:** Mohammed A Bafail, AbdullabAli PeerZada, Rajeh Alrajeh, Faisal M Alseraya, Haya S AlJurayb

**Affiliations:** 1 Pathology and Clinical Laboratory Medicine, King Fahad Medical City, Riyadh, SAU; 2 Laboratory Medicine, King Fahad Medical City, Riyadh, SAU

**Keywords:** acute myeloid leukemia, bcr::abl1 fusion, bone marrow transplant, chimerism, minimal residual disease

## Abstract

We recently reported a de novo acute myeloid leukemia (AML) patient harboring both BCR::ABL1 p190 isoform and RUNX1::MECOM fusion, a rare and high-risk molecular profile. In this follow-up, we present the patient’s post-transplant course with serial minimal residual disease (MRD) monitoring. MRD was tracked via quantitative polymerase chain reaction (qPCR) for the p190 isoform, and chimerism was assessed using short tandem repeat-polymerase chain reaction with capillary electrophoresis. The patient underwent haploidentical bone marrow transplantation after standard induction therapy complicated by sepsis and myocarditis. Post-transplant recovery was marked by poor initial engraftment, requiring platelet transfusions and biweekly filgrastim. A CD34+ boost on day +63 improved platelet counts and eliminated transfusion dependence by day +103. Chimerism studies showed a decline in donor DNA from 100% on days +30 and +60 to 86% by day +180, with CD3-positive donor cells at 72% on day +189. Despite hematologic recovery, qPCR consistently revealed persistent BCR::ABL1 p190 expression, indicating residual disease. This case underscores the challenge of eradicating leukemic stem cells (LSCs) in high-risk AML and supports the integration of next-generation flow cytometry for enhanced MRD and LSC assessment post-transplant.

## Introduction

Acute myeloid leukemia (AML) with BCR::ABL1 fusion is a rare but high-risk entity characterized by aggressive disease progression and poor response to conventional therapies. The presence of BCR::ABL1 in AML is associated with adverse clinical outcomes, largely due to the fusion gene’s ability to activate proliferative and resistance pathways [1]. The coexistence of additional genetic abnormalities, such as RUNX1::MECOM rearrangements, further complicates prognosis and therapeutic decision-making [2]. For such high-risk AML cases, allogeneic hematopoietic stem cell transplantation remains the most effective curative strategy, with long-term success heavily reliant on the eradication of minimal residual disease (MRD). Persistent MRD following transplant is strongly associated with relapse and poor survival, underscoring the importance of longitudinal molecular monitoring and individualized post-transplant strategies [3,4].

We previously described a de novo AML case with coexisting BCR::ABL1 p190 and RUNX1::MECOM rearrangements, highlighting the rarity of this molecular profile [5]. In this follow-up report, we present the patient’s post-transplant clinical course, with a focus on serial MRD and chimerism monitoring. The objective is to illustrate the challenges associated with persistent MRD despite hematologic remission and to emphasize the utility of integrated molecular surveillance tools. This case contributes to the limited body of literature on longitudinal post-transplant outcomes in pediatric BCR::ABL1 p190-positive AML, particularly in the setting of dual high-risk fusions.

## Case presentation

Case history

The patient, a 13-year-old female, was diagnosed with AML with monocytic differentiation, confirmed by morphology, immunophenotyping, fluorescence in situ hybridization, and next-generation sequencing (NGS). We previously reported the case [5].

Differential diagnosis, investigations, and treatment

Differential diagnosis was blast crisis chronic myeloid leukemia (CML) and AML, with the final diagnosis of AML having BCR::ABL1 fusion, given more than 20% blasts expressing a myeloid immunophenotype in the bone marrow or peripheral blood [6]. Initial treatment included cytarabine, daunorubicin, and etoposide (ADE) induction, which was complicated by episodes of sepsis, myocarditis, and admissions to the intensive care unit. The patient achieved complete remission with negative MRD before transplant. Given the high-risk molecular profile, she underwent haploidentical bone marrow transplantation (BMT) from her sister on May 14, 2024, following conditioning with busulfan, fludarabine, and antithymocyte globulin. The donor, her HLA-haploidentical sister, was sex-matched (female) and blood group O positive, while the recipient was AB positive. Both the donor and recipient were seropositive for cytomegalovirus and herpes simplex virus before transplantation.

Outcome and follow-up post-transplant monitoring

**Figure 1:**
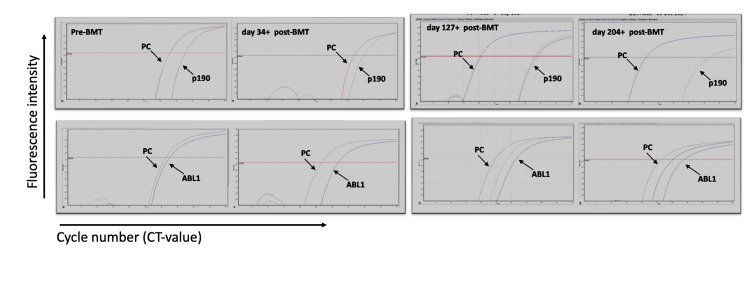
MRD monitoring through qPCR amplification of BCR::ABL1 p190 (top panel) and ABL1 (bottom panel) before and after BMT. qPCR is performed for BCR::ABL1 p190 using Ipsogen kits on the RotorGene instrument using the standard curve method for copy number calculation. NCN is calculated by taking a ratio of p190/ABL copy number, and the results are reported as NCN%. Different time points represent post-transplant MRD monitoring. MRD: minimal residual disease; qPCR: quantitative polymerase chain reaction; BMT: bone marrow transplantation; NCN: normalized copy number

The patient achieved hematologic recovery following BMT, although qPCR monitoring consistently demonstrated persistent BCR-ABL1 p190 positivity. Serial quantitative polymerase chain reaction (qPCR) results are presented in Table 1, highlighting ongoing MRD despite hematologic improvement. Representative amplification curves from selected time points are shown in Figure 1, illustrating persistent BCR-ABL1 p190 expression. A CD34+ cell boost was administered on day +63, resulting in improved platelet counts and absolute neutrophil count (ANC) stability. Key hematologic parameters post-CD34+ infusion are summarized in Table 2, showing progressive recovery in white blood cell (WBC) count, ANC, hemoglobin, and platelets. Engraftment was initially suboptimal, requiring regular platelet transfusions and biweekly filgrastim to maintain an ANC above 500/µL. Filgrastim was discontinued on day +78. Platelet transfusion dependence resolved after day +103.

**Table 1:** MRD monitoring by qPCR for BCR::ABL1 p190 isoform post-BMT. MRD: minimal residual disease; BMT day #: number of days post-bone marrow transplant; qPCR: quantitative polymerase chain reaction; NCN: normalized copy number

BCR::ABL1 p190 isoform qPCR		
BMT day #	BCR::ABL1/ABL1 (qPCR)	NCN (%)
0 (diagnosis)	0.9	90
+34	0.000085	0.0085
+104	0.00015	0.015
+127	0.00143	0.1437
+142	0.000106	0.0107
+177	0.007	0.7
+204	0.00056	0.0568

**Table 2:** CBC parameters at key time points post-BMT and CD34+ infusion. BMT day #: number of days post-bone marrow transplant; WBC: white blood cell count; ANC: absolute neutrophil count

BMT day #	WBC (×10³/µL) (reference = 4.0–11.0)	ANC (×10³/µL) (reference = 1.5–7.0)	Hemoglobin (g/dL) (reference = 12–16)	Platelet count (×10³/µL) (reference = 150–400)	Notes
+63	1.4	0.34	7.8	10	Pre-CD34+ cell infusion
+85	2.3	1.4	9.7	25	Post-CD34+ (day +22 post-infusion)
+103	3.1	2.2	8.6	49	Achieved transfusion independence
+200	5.7	2.9	9.2	145	Late follow-up, stable counts
+240	4.78	1.81	10.7	131	The patient remained stable without overt signs of relapse

Chimerism testing revealed full donor engraftment early post-transplant, with a gradual shift toward mixed chimerism over time. A transient improvement in donor percentage was noted mid-course, followed by recurrent fluctuations. Detailed chimerism trends are summarized in Table 3.

**Table 3:** Post-transplant chimerism results over time. BMT day #: number of days post-bone marrow transplant

BMT day #	Donor DNA (%)	Patient DNA (%)
+33	100	0
+58	100	0
+90	99	1
+126	97	3
+176	86	14
+203	91	9
+212	84	16
+245	92	8
+280	92	8
+309	90	10
+330	84	16

The most recent complete blood count on day +240 showed a WBC count of 4.78 × 10³/µL, ANC of 1.81 × 10³/µL, lymphocytes at 2.04 × 10³/µL, red blood cell count at 3.10 × 10⁶/µL (low), hemoglobin of 10.7 g/dL (low), and platelets at 131 × 10³/µL (low). Clinically, the patient remained stable without overt signs of relapse.

## Discussion

MRD following allogeneic transplantation remains a major clinical challenge in high-risk AML. We describe a pediatric de novo AML case with BCR::ABL1 (p190) and RUNX1::MECOM fusions, demonstrating sustained MRD positivity post-transplant. This case underscores the difficulty of achieving full leukemic eradication and raises the possibility of leukemic stem cell (LSC) persistence. While not performed, next-generation flow cytometry (NGF) may offer future utility for MRD and LSC assessment in such settings.

This case report highlights the clinical and molecular complexity of managing high-risk de novo AML with persistent MRD following allogeneic stem cell transplantation. Although MRD persistence is a well-established predictor of relapse in AML, few reports have longitudinally assessed MRD in BCR::ABL1-positive AML using the p190 isoform. Our case represents a rare pediatric example with coexisting BCR::ABL1 p190 and RUNX1::MECOM fusions, monitored post-transplant with serial qPCR and chimerism analysis to track disease burden and engraftment.

Allogeneic BMT remains the most effective curative strategy for patients with high-risk AML or adverse cytogenetics and molecular abnormalities such as BCR::ABL1 and, in fact, may provide superior survival outcomes compared to chemotherapy alone [7]. The residual leukemic cells are eliminated through the graft-versus-leukemia effect, mediated by donor immune cells [8]. In a systematic review and meta-analysis by Li et al., BMT showed reduced relapse rates with improved long-term survival, suggesting its efficacy as a key therapeutic modality [9]. Despite its better clinical outcome, BMT has its own risks and limitations, especially in pediatric and high-risk patients. Complications include poor graft function necessitating a CD34+ cell boost to support engraftment [10], as seen in our patient, who developed severe neutropenia and remained platelet transfusion dependent. The CD34+ infusion was administered to enhance marrow recovery and promote hematopoietic reconstitution in the setting of delayed count recovery, despite early full donor chimerism. Additionally, BMT may be associated with cardiovascular, pulmonary, hepatic, endocrine, and skeletal disorders, as well as infertility and iron overload [11].

From a laboratory perspective, both chimerism and MRD analyses are essential components of post-transplant monitoring, offering early insight into engraftment quality and relapse risk. Complete donor chimerism typically reflects successful graft establishment, whereas mixed chimerism, marked by the coexistence of donor and recipient hematopoiesis, has been associated with an elevated risk of relapse in AML [12]. In our patient, donor chimerism gradually declined from 100% at early post-transplant time points to 86% by day +176, followed by a transient increase to 92% after CD34+ cell infusion, suggesting a partial recovery of donor dominance. These dynamics mirror findings in recent studies linking early mixed chimerism to inferior survival outcomes, even when subsequent donor recovery occurs [12]. As such, serial chimerism monitoring remains a valuable prognostic tool and supports timely interventions when declining donor percentages are observed, regardless of hematologic stability.

MRD monitoring is a highly sensitive tool that enables the detection of leukemic cells below the threshold of morphologic assessment and facilitates timely therapeutic intervention [13]. Persistent MRD following allogeneic transplantation has consistently been associated with inferior outcomes, including higher relapse rates and reduced overall survival [14]. In contrast, patients who achieve MRD negativity post-transplant experience significantly improved relapse-free and overall survival [15]. While established molecular MRD markers, such as *NPM1* mutations and RUNX1::RUNX1T1, CBFB::MYH11, and PML::RARA fusions, are widely used in AML [16], there is currently no standardized or validated qPCR assay available for monitoring RUNX1::MECOM rearrangements [17], and thus, MRD tracking for this target was not pursued in our case. For BCR::ABL1-positive cases, particularly those expressing the p190 isoform, MRD tracking using qPCR is routinely applied in CML and was adapted here as a surrogate marker in this high-risk AML setting [18]. In our patient, serial qPCR consistently detected the p190 isoform post-transplant, indicating persistent leukemic burden and suggesting the presence of a chemotherapy-resistant LSC clone.

NGF has emerged as a powerful tool for MRD detection in AML, with higher sensitivity than conventional flow cytometry and broader applicability than molecular assays alone. Recent studies show that NGF enables detection of MRD at levels below 0.01%, correlating with post-transplant outcomes and providing earlier relapse risk stratification [19]. Additionally, peri-transplant assessment using NGF and NGS has also been shown to improve prognostic accuracy and guide therapeutic decisions in AML undergoing allogeneic transplantation [20]. Given these advantages, NGF may be especially valuable in high-risk AML with complex clonal architecture, such as our case, and warrants consideration in post-transplant surveillance strategies.

Despite these strategies, persistent MRD remains a significant challenge. While our patient achieved favorable hematologic outcomes post-BMT, the continued presence of MRD highlights the complexity of treating high-risk AML.

## Conclusions

This case underscores the clinical challenge of managing high-risk de novo AML with dual genetic fusions, BCR::ABL1 p190 and RUNX1::MECOM. Despite hematologic remission post-transplant, persistent MRD and declining donor chimerism revealed ongoing disease activity. These findings highlight the importance of serial molecular and chimerism monitoring in post-transplant care and support the use of advanced tools such as NGF to detect residual LSCs. Tailored molecular surveillance and treatment strategies are essential to optimize outcomes in such high-risk pediatric AML.
